# Intestinal barrier dysfunction: an evolutionarily conserved hallmark of aging

**DOI:** 10.1242/dmm.049969

**Published:** 2023-05-04

**Authors:** Anna M. Salazar, Ricardo Aparicio, Rebecca I. Clark, Michael Rera, David W. Walker

**Affiliations:** ^1^ Department of Molecular Biology and Chemistry, Christopher Newport University, Newport News, VA 23606, USA; ^2^ Department of Integrative Biology and Physiology, University of California, Los Angeles, CA 90095, USA; ^3^ Department of Biosciences, Durham University, Durham DH1 3LE, UK; ^4^ Université de Paris, Inserm U1284, Center for Research and Interdisciplinarity, Paris 75004, France; ^5^ Molecular Biology Institute, University of California, Los Angeles, CA 90095, USA

**Keywords:** Aging, Intestine, Pathology

## Abstract

A major challenge in the biology of aging is to understand how specific age-onset pathologies relate to the overall health of the organism. The integrity of the intestinal epithelium is essential for the wellbeing of the organism throughout life. In recent years, intestinal barrier dysfunction has emerged as an evolutionarily conserved feature of aged organisms, as reported in worms, flies, fish, rodents and primates. Moreover, age-onset intestinal barrier dysfunction has been linked to microbial alterations, elevated immune responses, metabolic alterations, systemic health decline and mortality. Here, we provide an overview of these findings. We discuss early work in the *Drosophila* model that sets the stage for examining the relationship between intestinal barrier integrity and systemic aging, then delve into research in other organisms. An emerging concept, supported by studies in both *Drosophila* and mice, is that directly targeting intestinal barrier integrity is sufficient to promote longevity. A better understanding of the causes and consequences of age-onset intestinal barrier dysfunction has significant relevance to the development of interventions to promote healthy aging.

## Teaser

**Summary:** Studies in model organisms have shown that age-onset intestinal barrier dysfunction is linked to systemic health decline. Targeting intestinal barrier integrity is, therefore, a promising approach to prolong health span.

## Introduction

**Fig. 1.**
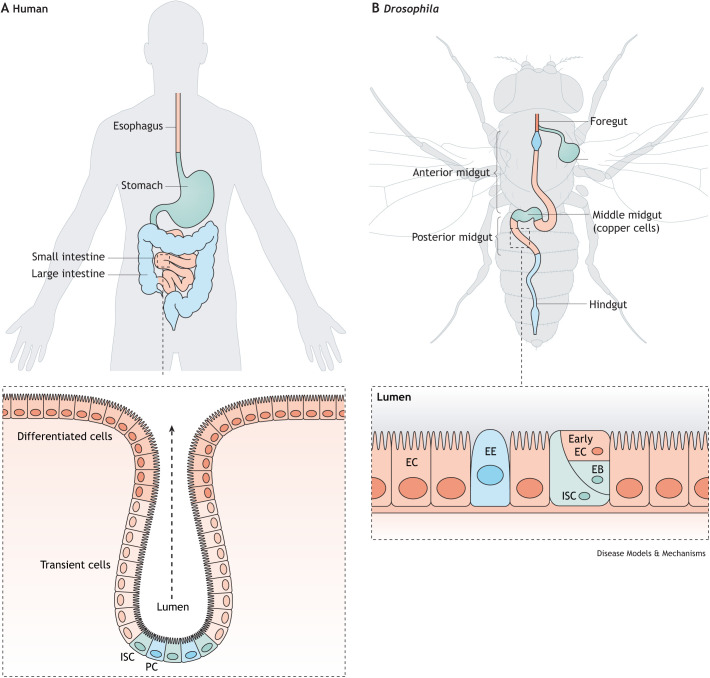
**Overview of *Drosophila* and human digestive tracts.** (A) The mammalian intestine retains fundamental processes important for stem cell renewal, epithelial cell regeneration and epithelial cell interaction with the microbiota. Mammalian ISCs divide to self-renew and to form transient amplifying cells that undergo rounds of division as they migrate away from the ISCs and differentiate into ECs, EEs and goblet cells that secrete mucin. PCs are positioned in the crypt with ISCs and are important for producing AMPs to maintain microbial homeostasis. (B) The *Drosophila* midgut is simpler than the mammalian intestine and is comprised of ISCs that divide to form more ISCs and EBs. EBs differentiate to form ECs, the absorptive cells in the gut, and the secretory EEs. AMP, antimicrobial peptides; EB, enteroblast; EC, enterocyte; EE, enteroendocrine cell; ISC, intestinal stem cell; PC, Paneth cell.

The intestinal barrier has evolved as a multilayer system, providing a physical as well as a functional barrier against harmful substances entering the body (Ghosh et al., 2020; Vancamelbeke and Vermeire, 2017). These interdependent layers include the luminal mucus layer, the gut epithelial layer, and a third, internal layer that forms the mucosal immune system (France and Turner, 2017). In humans, the gut epithelial layer is formed of a continuous sheet of epithelial cells, including enterocytes (see Glossary, Box 1), intestinal stem cells (ISCs; Box 1), enteroendocrine cells (Box 1), goblet cells and Paneth cells (Fig. 1A). Goblet cells produce mucus and Paneth cells produce antimicrobial peptides (AMPs), which are released into the luminal mucus layer to maintain microbial homeostasis. The epithelial cells are tightly attached to each other by junctional complexes, which prevent bacteria or other solutes from traversing the epithelial layer, where they would encounter various cell types from the mucosal immune system and potentially trigger systemic responses.

**Box 1. Glossary**

**Adherens junctions:** protein complexes located between adjacent epithelial cells that are important for initiating and mediating the maturation and maintenance of contact between adjacent cells.

**Autophagy:** a process whereby cellular materials are sequestered within double-membrane vesicles, known as autophagosomes, and delivered to the lysosome for degradation.

**Bacterial homogenates:** homogenates obtained by surface sterilizing flies with 70% ethanol to ensure that only internal microbes are present, then homogenizing with a motor pestle in sterile buffer prior to addition to the fly food, to reintroduce bacteria to axenic, or germ-free, organisms.

**Dietary restriction:** reduction in nutrient intake, for example reducing yeast content in the prepared laboratory food for *Drosophila*.

***Drosophila* peroxisome proliferator-activated receptor gamma coactivator 1 (*PGC-1*) homolog (*dPGC-1*):** a transcriptional coactivator that regulates genes involved in energy metabolism, including mitochondrial biogenesis and respiration.

**Dysbiosis:** an imbalance in the proportions of bacterial populations, or changes in bacterial loads.

**Enterocytes:** intestinal epithelial cells involved in absorption of ions, nutrients, water, etc.

**Enteroendocrine cells:** specialized cells found in the stomach, gastrointestinal tract and pancreas that secrete hormones in response to luminal contents that regulate food intake, digestion and intestinal motility, and can recognize and react to pathogens and microbes, including commensal bacteria.

**Gut transit time:** the time it takes for the movement of luminal content through the gastrointestinal tract – a marker of gut health, function and motility, with links to host and microbiome metabolism.

**Hallmark of aging:** 12 hallmarks of aging common in numerous organisms have been proposed and include genomic instability, telomere attrition, epigenetic alterations, loss of proteostasis, disabled macroautophagy, deregulated nutrient-sensing, mitochondrial dysfunction, cellular senescence, stem cell exhaustion, altered intercellular communication, chronic inflammation and dysbiosis.

**Hemolymph:** arthropod circulatory system, analogous to vertebrate blood, composed of fluid plasma and hemocytes, which are immune cells in invertebrates.

**Immunoglobulin A:** the most abundant antibody isotype produced at mucosal surfaces, including the intestines, that provides protection against pathogens and toxins, with high levels indicating an elevated immune response.

**Inflammaging:** chronic, sterile, low-grade inflammation associated with aging.

**Inflammation:** the recruitment of immune cells and the release of peptides and chemicals to target and impair pathogens in mammals and invertebrates.

**Insulin/insulin-like growth factor 1 (IGF-1):** peptide hormones with similar molecular structures that are involved in growth and metabolism through promoting absorption of glucose by liver, muscle, fat cells and protein synthesis, with IGF-1 mediating the effects of growth hormones to promote cell growth and division.

**Intestinal barrier dysfunction:** decline of intestinal barrier integrity characterized by increased permeability, or a ‘leaky gut’.

**Intestinal lumen:** the interior of the intestinal tract.

**Intestinal stem cells (ISCs):** adult multipotent cells that continually self-renew through cell division and differentiate into the specialized intestinal cells located in the intestinal epithelium.

**Lipopolysaccharide (LPS):** large lipid and polysaccharide molecules, considered bacterial toxins, located on the outer membrane of gram-negative bacteria that potently activate immune function in multiple organisms, including mammals and invertebrates.

**Mitophagy:** the autophagic removal of damaged or superfluous mitochondria, via mitochondrial autophagy.

**Multi-sugar test:** the simultaneous ingestion of sucrose, lactulose, mannitol and sucralose in order to test permeability of the gastroduodenum, small intestine and large intestine, respectively, through measuring the urine before and after sugar consumption. This is a non-invasive alternative to the Smurf assay that can be utilized in mammals, including humans.

**Smurf flies/Smurf assay:** *Drosophila melanogaster* that have consumed a non-absorbable blue dye that has translocated from the gut into the *Drosophila* circulatory system, thereby causing the entire fly to turn blue. This assay is a non-invasive way to determine intestinal barrier dysfunction.

**Terminal ileum:** the last section of the small intestine prior to the cecum of the large intestine.

**Tight junction/septate junction:** protein complexes that prevent the movement of ions, solutes and water between adjacent epithelial cells in the gut and are critical for intestinal barrier permeability, also called occluding junctions, with invertebrate equivalents termed septate junctions**.**

**Zonulin:** the mammalian analog of the zonula occludens toxin that increases the permeability of tight junction proteins between adjacent intestinal epithelial cells and is secreted by the bacterial pathogen that causes cholera. Increased levels of zonulin signaling are associated with intestinal permeability and observed in response to gliadin, a glycoprotein found in gluten.

Studies in diverse species have reported that age-onset intestinal barrier dysfunction (Box 1) is a shared pathophysiological hallmark of aging (Box 1; Martins et al., 2018). Indeed, loss of intestinal barrier function has been reported in aged worms (Dambroise et al., 2016; Egge et al., 2019; Gelino et al., 2016), flies (Dambroise et al., 2016; Rera et al., 2011, 2012), fish (Dambroise et al., 2016), rodents (Katz et al., 1987; Kühn et al., 2020; Ma et al., 1992; Thevaranjan et al., 2017), monkeys (Kavanagh et al., 2016; Mitchell et al., 2017) and humans (Kavanagh et al., 2019; Liu et al., 2020). Moreover, an emerging theme from these studies is that impaired intestinal barrier function in aged organisms is tightly linked to systemic health decline.

The major goal of this Review article is to provide a comprehensive overview of research into the role of intestinal barrier dysfunction in organismal aging. Studies in the fruit fly, *Drosophila melanogaster*, were instrumental in allowing fundamental questions in the field to be addressed (Clark et al., 2015; Rera et al., 2011, 2012, 2013b). Since then, significant progress has been made in our understanding of the molecular and cellular mechanisms that maintain the intestinal barrier during aging (Clark et al., 2015; Egge et al., 2019; Resnik-Docampo et al., 2017, 2018; Salazar et al., 2018). Furthermore, it has become apparent that intestinal barrier dysfunction is linked to both microbial dysbiosis (Box 1) and systemic inflammation (Box 1) in flies and mammals (Clark et al., 2015; Clark and Walker, 2018; Keebaugh and Ja, 2017; Mitchell et al., 2017; Rera et al., 2012; Salazar et al., 2018; Thevaranjan et al., 2017). The role of intestinal barrier dysfunction in disease has been discussed at length (Lewis and Taylor, 2020; Odenwald and Turner, 2017; Turner, 2009; Vancamelbeke and Vermeire, 2017) and is not a focus of this Review.

Finally, we discuss recent data showing that interventions targeting intestinal barrier integrity can prolong lifespan in both *Drosophila* and mice (Kühn et al., 2020; Salazar et al., 2018). Taken together, these findings support the idea that loss of intestinal barrier function is a key event that drives systemic aging.

### Intestinal barrier integrity is tightly linked to systemic aging phenotypes and lifespan determination in *Drosophila*

*Drosophila* are a tractable genetic model system that is well suited to identify the broad principles of senescent pathophysiology that underlies age-onset health decline. As outlined in Fig. 1, there are a number of similarities between fly and human intestines, which led to the emergence of two research themes in the *Drosophila* aging field in the late 2000s and early 2010s that were particularly informative. The first area of research focused on characterizing age-related changes in the intestinal epithelium at the cellular level. In this work, it was shown that there is a dramatic age-related increase in ISC proliferation, which is accompanied by an accumulation of mis-differentiated daughter cells that express markers of both ISCs and terminally differentiated daughter cells, leading to perturbed ISC function (Jasper, 2015). These studies provided clear evidence of age-onset intestinal degeneration that would likely impact intestinal function and organismal health. However, although ISCs of the fly midgut closely resemble stem cell populations in mammals (Apidianakis and Rahme, 2011), we are still far from having a complete understanding of how aging impacts ISCs across taxa.

The second area of research focused on the role of the intestine as a key target organ for a number of genetic interventions that prolong lifespan (Biteau et al., 2010; Guo et al., 2014; Hur et al., 2013; Rera et al., 2013a, 2011; Ulgherait et al., 2014). Taken together, these studies strongly supported the concept that intestinal homeostasis is a key component of fly longevity. However, a number of questions remained unanswered, including what are the key functional defects that occur in the aged intestine and what is the relationship between intestinal pathophysiology and age-onset mortality.

A key experimental advance that has greatly improved our understanding of the role of intestinal homeostasis in organismal aging was the development of non-invasive approaches to monitor intestinal barrier function in living flies (Martins et al., 2018; Rera et al., 2011, 2012). More specifically, these assays rely upon non-absorbable dyes to identify flies with intestinal barrier dysfunction, via the presence of the dye outside of the digestive tract and throughout the body. Because the dye most commonly used in such assays is a blue food dye, flies showing intestinal barrier dysfunction have been referred to as ‘Smurf flies’ and the assay itself as the ‘Smurf assay’ (Box 1) (Martins et al., 2018; Rera et al., 2011, 2012). The first description of the Smurf assay was in a study showing that upregulation of the *Drosophila* peroxisome proliferator-activated receptor gamma coactivator 1 (*PGC-1*) homolog (*dPGC-1*; Box 1) in ISCs and progenitor cells within the digestive tract extends lifespan, which was linked to improved intestinal barrier function (Rera et al., 2011). This was the first indication that age-onset intestinal barrier dysfunction may be linked to organismal lifespan. However, at the time, the relationship between intestinal barrier integrity and organismal health in individual flies was not known.

One year later, the Smurf assay was used to further explore the role of intestinal barrier dysfunction in fly aging across a range of *Drosophila* laboratory strains and environmental conditions (Rera et al., 2012). Interventions that extend fly lifespan, such as reduced temperature or dietary restriction (Box 1), delay the onset of intestinal barrier dysfunction, whereas mitochondrial dysfunction accelerates the onset of intestinal barrier defects and shortens the lifespan. But, perhaps the most important finding that was published in this study was the link between the Smurf phenotype and age-onset mortality. By separating Smurf flies by chronological age, it was shown that intestinal barrier dysfunction is a better predictor of mortality than chronological age. Indeed, regardless of chronological age, flies that show intestinal barrier dysfunction had a significantly shorter remaining lifespan than chronologically age-matched non-Smurf flies. Moreover, this study also reported that intestinal barrier dysfunction was linked to additional markers of health decline, including markers of systemic inflammation, metabolic defects and behavioral decline (Rera et al., 2012). This research then formulated the idea that the physiological events that follow intestinal barrier dysfunction represent a distinct phase of aging (Clark et al., 2015; Tricoire and Rera, 2015). We will discuss these findings relating to systemic markers of aging (Lopez-Otin et al., 2023), with a focus upon inflammation, in detail below.

It should be noted, however, that a report has questioned the generality of the Smurf assay to be able to predict age-onset mortality (Bitner et al., 2020). One of the main claims of this work was that only a fraction of flies display the ‘Smurf phenotype’ upon death. However, there are many questions relating to the ability to score the Smurf phenotype under the conditions described, which include maintaining flies individually under atypical husbandry conditions. Indeed, the Smurf assay relies upon animals continuing to consume the blue dye under the experimental conditions tested. If a fly were to cease food or water intake for a period of time, for any reason, then it would be impossible to observe the Smurf phenotype. This is a limitation of the use of food dyes to probe intestinal integrity that should be considered.

### Intestinal barrier dysfunction is an evolutionarily conserved hallmark of aging

Although the work described above in the *Drosophila* model was the first to characterize the impact of intestinal barrier perturbation on systemic aging phenotypes and lifespan determination, there had been prior reports of intestinal barrier impairments in aged animals. Indeed, pioneering work in rodents provided early insight into age-related alterations in intestinal permeability (Branca et al., 2019). More specifically, a number of studies using aged rats gavaged with different-sized permeability probes reported findings indicative of increased intestinal permeability with age (Katz et al., 1987; Ma et al., 1992). In a more recent study, intestinal barrier function was measured in mice at different ages (3, 12, 15 and 18 months), using fluorescein isothiocyanate (FITC)-labeled dextran and measuring translocation of fluorescence from the intestine into the blood plasma following oral feeding (Thevaranjan et al., 2017). Using this approach, intestinal permeability was shown to increase with age starting at around 15 months. Consistent with evidence of increased permeability in the colon, where bacterial numbers are highest, levels of the bacterial cell wall component, muramyl dipeptide, were also significantly higher in the plasma of old mice compared to that of young mice (Thevaranjan et al., 2017).

A series of studies also investigated the impact of aging on intestinal barrier function in various primate species, including humans (Branca et al., 2019). Markers of intestinal permeability, such as the amount of horseradish peroxidase that traversed the epithelial barrier, were reported to be significantly higher in colonic biopsies collected from old baboons compared with those from young baboons (Tran and Greenwood-Van Meerveld, 2013). These changes were linked to remodeling of intestinal epithelial tight junction (Box 1) proteins in colonic tissue from old baboons. In addition, three related studies in vervet monkeys have reported increased intestinal permeability with age (Kavanagh et al., 2016; Mitchell et al., 2017; Wilson et al., 2018). These studies detected an intestinal mucosal permeability marker, FITC-Dextran 40, in plasma samples of old, but not young, vervet monkeys. Moreover, the loss of intestinal barrier integrity was linked to sedentary behavior in individual aged monkeys (Kavanagh et al., 2016). However, the question of whether intestinal barrier dysfunction is a common feature of human aging remains inconclusive (Branca et al., 2019; Mabbott, 2015). The levels of serum zonulin (Box 1), an indirect marker of intestinal permeability, have been reported to be higher in healthy elderly people than in healthy young people (Qi et al., 2017), and colonic tissue from old healthy people showed alterations in intestinal epithelial tight junction proteins (Liu et al., 2020). In addition, a number of markers of intestinal barrier dysfunction were reported to be elevated in older adults with cardiometabolic disease compared to healthy aged-matched participants (Kavanagh et al., 2019). Examining biopsy tissues from human terminal ileum (Box 1), one study reported that intestinal permeability to solutes, but not macromolecules, was significantly increased in the intestines of elderly humans (Man et al., 2015). In conjunction, however, a recent study failed to detect a difference in intestinal permeability between healthy older people (65–75 years) and young people (18–40 years) utilizing a ‘multi-sugar test’ (Box 1) (Wilms et al., 2020). Additional work will be required to provide a clear understanding of the relevance of age-onset intestinal barrier dysfunction in human aging.

As noted above, the Smurf assay in *Drosophila* allowed an analysis of the impact of intestinal barrier perturbation on lifespan determination (Rera et al., 2011, 2012). This assay has since been adapted to detect intestinal barrier dysfunction in aged individuals from two other *Drosophila* species, *Drosophila mojavensis* and *Drosophila virilis*, the nematode *Caenorhabditis elegans* and, also, the vertebrate zebrafish *Danio rerio* (Dambroise et al., 2016). Importantly, this work also reported that the proportion of animals showing intestinal barrier dysfunction increases with age and is linked to mortality in each of the species. Additional studies have also reported loss of intestinal barrier integrity in aged worms using different technical approaches, including non-absorbable dyes (Egge et al., 2019; Gelino et al., 2016).

**Fig. 2.**
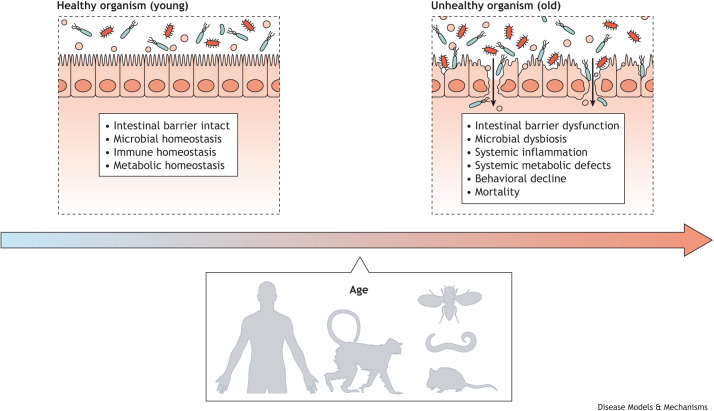
**Intestinal barrier dysfunction is an evolutionarily conserved hallmark of aging.** Organisms from *C. elegans* to humans exhibit microbial dysbiosis, inflammation, metabolic dysfunction and impending death, accompanying loss of intestinal barrier function with age.

In summary, it is now clear that impaired intestinal barrier integrity is an evolutionarily conserved pathophysiological feature of the aging process (Fig. 2). In and of itself, the fact that intestinal barrier function declines with age is perhaps not surprising; aging is characterized by physiological decline. The next level of questions relate to how this (or any) specific pathophysiological marker of aging contributes to organismal health decline.

### Relationship between intestinal barrier dysfunction and age-onset inflammation

A central theme of the ‘biology of aging’ is that the chronic, low-grade inflammation, which has been called ‘inflammaging’ (Box 1) (Franceschi, 2007; Fulop et al., 2023), contributes to the pathogenesis of age-related diseases (Ferrucci and Fabbri, 2018; Franceschi and Campisi, 2014; Furman et al., 2019; Nikolich-Zugich, 2018). It is, therefore, of vital interest to better understand the causes of this inflammation and to seek out therapeutic avenues that can ameliorate, or even reverse, these effects. The innate immune system consists of rapid, non-specific responses to pathogens and is composed of physical, cellular and chemical defenses, many of which are conserved in organisms as diverse as *Drosophila*, mice, monkeys and humans. Major pathways controlling the *Drosophila* immune response to pathogenic bacteria involve the activation of the conserved Toll pathway, the immune deficiency (IMD) pathway and Dual oxidase (Duox), which releases high levels of reactive oxygen species. Both the Toll and IMD pathways activate distinct nuclear factor-κB-like (NFκB) transcription factors to release AMPs that can inhibit bacterial growth (Buchon et al., 2014). Owing to the conserved characteristics in these immune responses, *Drosophila* provide a tractable model for investigating the link between inflammation, dysbiosis, intestinal barrier function and disease. Interestingly, several studies have shown that aging flies constitutively activate the transcription factor Forkhead box, sub-group O (dFOXO) (Guo et al., 2014; Morris et al., 2012; Rera et al., 2012), which is normally induced in response to inhibition of the insulin/insulin growth factor-1 (IGF-1; Box 1) signaling pathway (Teleman, 2009), the pathway crucial for an organism's metabolic regulation in development, growth and longevity. Activation of dFOXO in intestinal enterocytes (Box 1) decreases Peptidoglycan recognition protein SC2 (PGRP-SC2) expression, leading to an activation of the innate immune response (Guo et al., 2014). Therefore, knocking out gut PGRP-SC2 results in an increase in dysbiosis, AMP expression and stem cell dysplasia, whereas overexpressing PGRP-SC2 has the inverse effect, revealing the connection between dFOXO expression and immune regulation in the aging gut (Guo et al., 2014).

Using the Smurf assay in *Drosophila*, the role of immune regulation in age-dependent loss of barrier function was investigated further. This method revealed that, as flies age, they lose barrier function, with a concomitant increase in systemic, whole-body inflammation prior to death (Rera et al., 2012). In order to understand the molecular correlates of the association between intestinal barrier function and mortality, the expression of immunity-related genes was examined to elucidate whether inflammation plays a role in the observed mortality of Smurf flies. In the fruit fly, an elevation of AMPs occurs in response to disruptions in the microbiome and is imperative for maintaining homeostasis in the microbiome. AMP gene expression was assessed by quantitative PCR in age-matched flies exhibiting barrier dysfunction, revealing that intestinal barrier permeability is accompanied by significantly increased levels of AMPs in the gut and systemically, regardless of chronological age (Rera et al., 2012). Both young and aged Smurf flies exhibit significantly higher levels of AMPs in comparison to those in age-matched non-Smurf flies, illustrating a possible explanation for previous data showing elevated AMPs in aged fly populations (Landis et al., 2004; Pletcher et al., 2002), because there is a higher proportion of Smurf flies as the flies age (Rera et al., 2012). Interestingly, aged flies without detectable barrier defects express similar AMP levels to those of young flies, implying the importance of barrier function in suppressing inflammation.

During aging, the microbiome undergoes dysbiosis and can activate inflammatory responses (Clark et al., 2015; Ren et al., 2007; Broderick et al., 2014; Guo et al., 2014). Studies in the fly have illustrated that intestinal barrier dysfunction is highly correlated with elevated bacterial levels in the gut, with Smurf flies showing increased bacterial levels regardless of chronological age (Clark et al., 2015; Rera et al., 2012). Lowering bacterial loads through antibiotic treatment or culturing flies in axenic, germ-free, conditions led to reductions in inflammation and delayed onset of intestinal barrier dysfunction (Clark et al., 2015). It is important to note, however, that these changes were reported in a specific laboratory strain, and it is likely that the relationships between commensal microbiota, intestinal homeostasis and lifespan may differ between laboratory strains. Nevertheless, these studies exemplify the cross-talk and close association between intestinal barrier function, inflammation and microbial homeostasis.

Furthermore, genetically inducing immune activation in the intestine/fat body of adult flies through the activation of the Toll receptor or the IMD pathway receptor led to a significant decrease in lifespan and a corresponding increase in Smurf flies, showing that the relationship between immune activation and intestinal barrier dysfunction is bi-directional (Clark et al., 2015). A related study has also reported that flies with a constitutively active gut immune system show an increase in bacterial levels and dysbiosis, but the study did not investigate the flies' intestinal barrier status (Dantoft et al., 2016). In addition, feeding young flies bacterial homogenates (Box 1) obtained from the guts of aged flies led to an increase in gut permeability and a shortened lifespan, indicating that initiating dysbiosis in young flies is sufficient to accelerate intestinal barrier dysfunction, leading to an earlier death (Clark et al., 2015). In these flies, systemic inflammation occurs rapidly and is observed 8 h post Smurf detection. Interestingly, treating flies with antibiotics within 24 h of detectable barrier dysfunction prevented post-Smurf dysbiosis, leading to a significant reduction in the activation of the innate immune response, with decreased expression of AMPs and a significantly extended lifespan, compared to those in Smurf flies not exposed to antibiotics. These data led to the conclusion that microbial dysbiosis, with the accompanying activation of inflammation, was the primary cause of death in this population of flies, not barrier dysfunction (Clark et al., 2015). Again, these effects were confirmed in a specific laboratory strain, and it is possible that different strains show distinct pathophysiologies in late life.

Inflammation accompanying barrier dysfunction is also a key feature in mammals, including mice, monkeys and humans. Indeed, it has been reported that, following age-onset intestinal barrier dysfunction, microbial products enter the bloodstream of aged mice, where they trigger systemic inflammation, as demonstrated by elevated levels of serum interleukin (IL)-6, among other indicators (Thevaranjan et al., 2017). Aging vervet monkeys exhibit intestinal barrier dysfunction, with microbial translocation across the intestines, into the blood plasma, increasing with age. An increase in bacterial levels and immune activation, with elevated plasma levels of immunoglobulin A (Box 1) and the AMP alpha defensin were observed in aged monkeys, showing a conservation between *Drosophila* and monkeys (Kavanagh et al., 2016; Wilson et al., 2018). In addition, human intestinal barrier dysfunction, determined by levels of microbial translocation markers, such as lipopolysaccharide (LPS; Box 1)-binding protein (LPB) levels in the blood plasma, was associated with impaired physical function and inflammation in healthy adults over 60 years of age (Stehle et al., 2012). Another study examined the role of intestinal barrier dysfunction in overweight or obese adults with health comorbidities, such as cardiovascular disease or cardiometabolic dysfunctions and self-reported mobility impairments. A significant correlation between microbial translocation and inflammatory markers such as IL-6 and IL-8 was observed (Kavanagh et al., 2019). Furthermore, diet and exercise interventions did not impact LPB levels, implying that barrier function was still impaired, emphasizing the importance of preventing intestinal barrier dysfunction as a therapeutic avenue (Kavanagh et al., 2019).

One of the most important conclusions from the studies discussed above has been the close connection between inflammaging and age-onset intestinal barrier dysfunction. Studies in diverse species have found that loss of intestinal barrier function during aging is linked to markers of inflammation, both within the gut and systemically. A challenge for future studies will be to better understand the temporal dynamics involved and also to better understand the potential role of inflammation in perpetuating intestinal barrier dysfunction.

### Cellular mechanisms that modulate age onset of intestinal barrier dysfunction

Identifying interventions that delay the onset of intestinal barrier dysfunction is an attractive approach to promote healthy aging and provide mechanistic understanding of the underlying pathophysiology. In this regard, several studies have focused upon manipulating various specific components of the diet and examining the impact on intestinal barrier function (Box 2) (Akagi et al., 2018; Fan et al., 2017; Galenza and Foley, 2021; Gelino et al., 2016; Pereira et al., 2018; Regan et al., 2016; Ren et al., 2021; Rera et al., 2012; Xie et al., 2020).

**Box 2. Dietary modulation of intestinal barrier dysfunction**

Modulating the diet can have a pronounced effect on intestinal barrier function, impacting aging and lifespan. Interestingly, the effects of high-sugar diets have been reported to produce seemingly contradictory effects on intestinal integrity in *Drosophila*. Whereas high-sucrose diets were reported to increase the fraction of flies showing intestinal barrier failure (Pereira et al., 2018), supplementing the diet with glucose led to improved intestinal barrier function and increased lifespan (Galenza and Foley, 2021). It is not clear what underlies these effects, but it may prove important to examine gut transit time (Box 1) under these conditions. In this way, it could be determined whether dietary interventions that slow gut transit time are linked to improved intestinal barrier function during aging. In related work in mice, it has been shown that feeding middle-aged mice a high-fat diet compromised the epithelial barrier function of the colon (Xie et al., 2020). However, one of the best studied interventions that can delay the onset of intestinal barrier dysfunction is dietary restriction (DR) – a regime that extends lifespan in many organisms. Indeed, various DR paradigms have been reported to improve intestinal barrier function in aged flies (Akagi et al., 2018; Regan et al., 2016; Rera et al., 2012), worms (Gelino et al., 2016), mice (Ren et al., 2021) and pigs (Fan et al., 2017). For example, in flies, at least, low-protein diets improve intestinal barrier integrity during aging (Akagi et al., 2018; Regan et al., 2016; Rera et al., 2012).

As dietary restriction (DR) has such a profound effect on intestinal barrier function and lifespan (Box 2), an obvious question arises: what is the cellular mechanism(s) by which DR maintains intestinal barrier integrity during aging? Several studies have shown that induction of the cellular recycling process, autophagy (Box 1), is an important component of DR-mediated longevity (Hansen et al., 2018). The role of autophagy in mediating intestinal barrier function during aging has been examined in worms and flies (Hansen et al., 2018). In worms, DR is linked to markers of elevated intestinal autophagy and autophagy gene activity is required in the intestine to preserve intestinal barrier integrity with age (Gelino et al., 2016). Key upstream regulators of autophagy include the nutrient sensors mTOR and AMP-activated kinase (AMPK), which are each highly conserved longevity determinants (Hansen et al., 2018). Intestinal overexpression of *AMPKalpha* (AMPK catalytic subunit, hereafter referred to as AMPK) in flies induces markers of autophagy and autophagy gene expression in the intestine (Ulgherait et al., 2014). Increased AMPK-mediated autophagy is linked to improved intestinal barrier function during aging and extended lifespan (Ulgherait et al., 2014). Rapamycin, a small molecule that acutely inhibits mTOR to induce autophagy can prolong lifespan in model organisms, including flies and mice (Bjedov et al., 2010; Dai et al., 2014; Flynn et al., 2013; Harrison et al., 2009; Johnson et al., 2013; Kennedy and Lamming, 2016; Majumder et al., 2012; Schinaman et al., 2019; Spilman et al., 2010; Wilkinson et al., 2012). Importantly, rapamycin treatment maintains intestinal barrier function during *Drosophila* aging (Schinaman et al., 2019), and functional autophagy is required for the rapamycin-mediated improvement in gut function in aged flies (Schinaman et al., 2019). Together, these findings support the idea that functional autophagy counteracts age-onset intestinal barrier dysfunction. However, the relevant autophagic cargo that is recycled to prevent intestinal barrier failure has yet to be established.

Interestingly, interventions that specifically induce mitophagy (Box 1) counteract intestinal barrier failure in aged flies (Aparicio et al., 2019; Rana et al., 2017; Schmid et al., 2022). As noted above, mitochondrial dysfunction leads to early-onset intestinal barrier failure (Rera et al., 2012). Hence, an emerging concept from these studies is that the accumulation of dysfunctional mitochondria in the aged intestine may drive intestinal barrier failure. First, as noted above, intestine-specific upregulation of the *Drosophila PGC-1* homolog, a key positive regulator of mitochondrial energy metabolism, maintains intestinal barrier function during aging (Rera et al., 2011). More recently, it has been reported that feeding flies or mice small molecules that suppress superoxide production from the mitochondrial electron transport chain can counteract diet-induced intestinal barrier dysfunction (Watson et al., 2021). Together, these findings strongly indicate that autophagy may counteract intestinal barrier dysfunction, via the removal of dysfunctional mitochondria.

It has also been shown that chronic activation of JAK/STAT signaling in the aged gut contributes to loss of intestinal barrier function and bacterial dysbiosis (Li et al., 2016). More specifically, the authors found that JAK/STAT pathway activity increases throughout the intestine of older flies. Genetic approaches that reduce JAK/STAT signaling specifically within the intestinal region containing copper cells can delay a number of age-related pathologies, including intestinal barrier dysfunction. Knockdown of different JAK/STAT components in adult flies also resulted in lower levels of gut bacteria and proliferating ISCs. Furthermore, knockdown of JAK/STAT components in the copper cell region confers an extended lifespan. An interesting area for future research is the potential interplay between autophagy and JAK/STAT signaling in intestinal homeostasis during aging.

A related goal of the field is to dissect the proximal cellular mechanisms that maintain the intestinal barrier during aging. Occluding junctions play critical roles in epithelial barrier function (Buckley and Turner, 2018; Tepass and Hartenstein, 1994). Alterations in intestinal epithelial junction expression and localization have been reported in aged flies (Clark et al., 2015; Resnik-Docampo et al., 2017; Salazar et al., 2018) and aged mammals (Meier and Sturm, 2009; Ren et al., 2014; Tran and Greenwood-Van Meerveld, 2013). In fact, mislocalization of gut epithelial junctional proteins and lowered expression of junctional mRNA levels are observed during midlife, even before detectable barrier dysfunction as assessed by the Smurf assay (Salazar et al., 2018). This junctional protein mislocalization, therefore, precedes detectable Smurf formation and the subsequent larger increases in bacterial loads and AMPs. These data imply that a better insight into the roles of junction proteins is imperative to understand the relationship between dysbiosis, barrier dysfunction and inflammation.

A recent study utilized an inducible tumor model in the *Drosophila* intestine to provide insight into the mechanisms of intestinal barrier failure in this context (Zhou and Boutros, 2020). In this work, the findings support a model whereby tumor-related JNK activation is responsible for septate junction protein disruption and intestinal barrier dysfunction, leading to commensal dysbiosis as well as immune activation. To explore the role of junction proteins further, a study in *Drosophila* has shown that intestine-specific knockdown of the tricellular junction protein Gliotactin in young flies can induce early-onset intestinal barrier dysfunction and markers of ISC deregulation, which also highlights the relationship between ISC homeostasis and intestinal barrier function (Resnik-Docampo et al., 2017).

A related study characterized the role of Snakeskin (Ssk), a septate junction-specific protein, in intestinal homeostasis in flies (Salazar et al., 2018). Intestinal knockdown of Ssk in adults leads to rapid-onset intestinal barrier dysfunction and death; therefore, to better understand the causal relationships between intestinal barrier failure and inflammation, Ssk was knocked down in young adult flies for 6–11 days. Ssk knockdown for 6 days led to a statistically significant reduction in AMPs and Duox in both Smurf and non-Smurf fly intestines, revealing that this alteration in innate immune responses occurs prior to detectable barrier dysfunction (Salazar et al., 2018). Interestingly, knockdown of Ssk for 7 days resulted in a significant elevation of bacterial load and dysbiosis, possibly due to earlier repression of intestinal AMPs and Duox (Salazar et al., 2018). Prolonged knockdown of Ssk for 11 days resulted in flies that exhibit an increase in both intestinal AMPs and Duox – most likely in response to the high bacterial loads – and display barrier dysfunction (Salazar et al., 2018). The results at day 11 were consistent with previous data in aged Smurf flies (Clark et al., 2015; Rera et al., 2012). Remarkably, restoration of Ssk in the young intestine reverses intestinal barrier failure, bacterial dysbiosis and lifespan, although elevation of several AMPs remained, perhaps to help maintain bacterial homeostasis. However, when Ssk knockdown experiments were conducted under antibiotic or axenic conditions, with intestinal bacterial and AMP levels not increasing, the lifespans of the Ssk knockdown flies were not improved, with the flies still displaying intestinal barrier dysfunction and dying rapidly. The simplest interpretation of these results is that Ssk knockdown flies do not die as a result of immune activation following dysbiosis, but it is also possible that the causes of death are different under each condition.

In *C. elegans*, there exists a single type of junction that serves both as an adherens junction (Box 1) and a tight junction (Armenti and Nance, 2012). It has been shown that age-related phosphorylation of a low-abundant actin variant, ACT-5, disrupts its interactions with adherens junction proteins (Egge et al., 2019). This, in turn, leads to rapid disorganization of the junction proteins and, hence, loss of intestinal barrier integrity in aged worms.

The work discussed above furthers our understanding of the cellular mechanisms that lead to age-onset intestinal barrier dysfunction. An ongoing priority is to leverage these findings to develop interventions that can maintain intestinal barrier integrity with age.

### Directly targeting the intestinal barrier to promote longevity

**Fig. 3.**
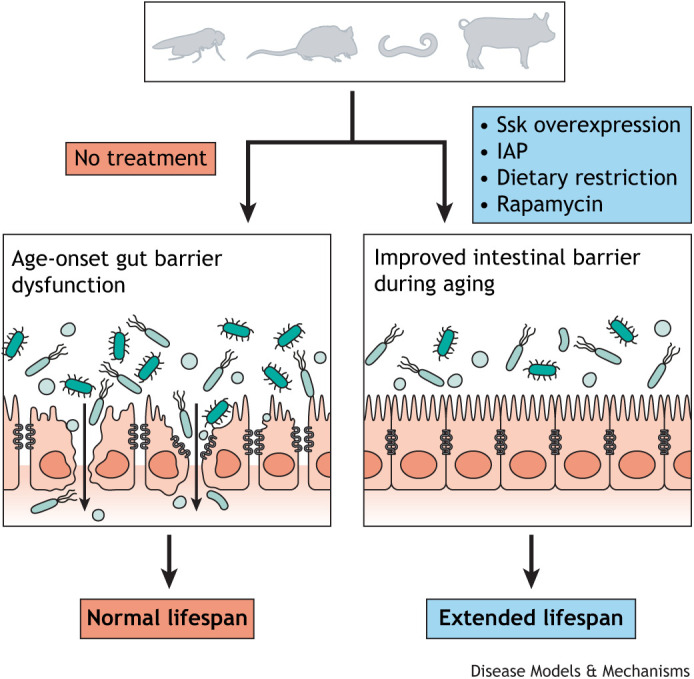
**Targeting intestinal barrier integrity to promote longevity.** Overexpression of intestinal junctional protein Ssk in flies, IAP supplementation in mice, dietary restriction in flies, worms, mice and pigs, and induction of autophagy with rapamycin in flies and mice lead to improved intestinal integrity and increased lifespans. IAP, intestinal alkaline phosphatase; Ssk, Snakeskin.

Intestinal barrier permeability is associated with dysbiosis and inflammaging, but whether maintaining gut barrier function with age could delay immune activation and improve health has not been clear until recently. Recent studies revealed therapeutic impacts accompanying an increase in intestinal barrier integrity (Fig. 3). In the same study exploring the impact of Ssk knockdown in young adult flies, Ssk was also overexpressed in *Drosophila* throughout their adult life using an inducible, gut-specific driver. Overexpression of Ssk led to improved intestinal barrier integrity in aged flies, with a highly significant reduction in Smurf flies observed in comparison to age-matched controls, alongside a significant decrease in age-associated dysbiosis and a modest, yet significant, increase in lifespan (Salazar et al., 2018). In addition, when fed pathogenic bacteria, *Serratia marcescens* Db11 (Nehme et al., 2007), flies overexpressing Ssk had a highly significant reduction in translocation of the pathogenic bacteria outside of the gut and into the hemolymph (Box 1), and had significantly improved survival compared to that of control lines (Salazar et al., 2018). Interestingly, there was a much more robust increase in lifespan observed in the Ssk-overexpressing flies, compared to that in controls, when flies were kept on a high-protein diet, most likely due to the detrimental effect of this diet on intestinal barrier function (Rera et al., 2012). Furthermore, the increase in lifespan observed with upregulated Ssk was eliminated when flies were fed antibiotics, or grown under germ-free, axenic conditions, implying that overexpressing Ssk promotes longevity by preventing microbial translocation (Salazar et al., 2018), consistent with previous results (Clark et al., 2015). Taken together, these data provided the first example of the extension of lifespan through targeting a junctional protein, leading to improved intestinal function, and suggests that future research into improved barrier function can lead to therapeutic breakthroughs.

Another study that explored a therapeutic role of improved intestinal barrier function in aging focused on the expression of intestinal alkaline phosphatase (IAP) (Kühn et al., 2020). In mammals, enterocytes secrete IAP into the intestinal lumen (Box 1) and bloodstream, where it acts on various substrates catalyzing the hydrolytic removal of phosphate groups (Singh and Lin, 2021). IAP is important in numerous physiological functions, including regulating pH and modulating long-chain fatty acids (Lallès, 2010, 2014; Singh and Lin, 2021). IAP is involved in decreasing inflammation in several ways. For instance, IAP dephosphorylates adenosine triphosphate (ATP), uridine diphosphate (UDP) and other pro-inflammatory signals, as well as LPS, which normally binds Toll-like receptor (TLR)4 in mammals to activate pro-inflammatory NFκB signaling. Dephosphorylation of LPS prevents its binding to TLR4 and the induction of the subsequent inflammatory responses that can lead to microbial dysbiosis, meaning that IAP also regulates commensal microbiota composition via LPS modulation (Lallès, 2010, 2014).

Most interestingly, recent evidence has emerged revealing that IAP can regulate tight junction expression levels. Mouse embryonic fibroblasts, derived from IAP knockout mice, have decreased expression of tight junction proteins zonula occludens (ZO)-1, ZO-2 and occludin, while systemic IAP knockout mice also show a reduction in the expression of ZO-1, ZO-2, occludin and claudin 1, accompanying a decrease in intestinal integrity (Hamarneh et al., 2014; Liu et al., 2016). In another study, IAP knockout in mice decreased lifespan, increased intestinal permeability and increased frailty, whereas IAP supplementation was shown to increase intestinal function, significantly extend the lifespan and decrease frailty (Kühn et al., 2020). Overexpressing IAP in human colon cell lines Caco 2 and T84 also resulted in upregulation of the tight junction components, Z0-1 and Z0-2, which improved barrier function and preserved tight junction localization (Liu et al., 2016). Furthermore, in *Drosophila*, a gut-specific double knockdown of the two analogous IAPs led to a decrease in mRNA expression of tight junction-equivalent proteins, E-cadherin (Ecad) and Discs large (Dlg), with a concomitant decrease in lifespan. The lifespan could also be partially restored by the addition of endogenous IAP in *Drosophila* (Kühn et al., 2020). It is worth noting that although the Toll pathway in *Drosophila* is similar to the mammalian TLR signaling pathway, it is activated by different stimuli, such as gram-positive bacteria and fungi, with LPS activating the IMD pathway in flies (Boutros et al., 2002). Overall, these studies emphasize the importance of tight junction components, inflammation and intestinal barrier function to overall health and lifespan and exemplify the therapeutic effect of IAP supplementation, which led to a decrease in age-associated inflammation and intestinal barrier dysfunction.

## Conclusions

The focus of this Review article is the importance of intestinal barrier function to organismal health and to our understanding of the mechanisms driving age-onset health decline. It has been possible to reveal that loss of intestinal barrier integrity is linked to multiple markers of systemic aging (Clark et al., 2015; Rera et al., 2012, 2013b; Salazar et al., 2018). An additional application of the Smurf assay is that it allows separation of the chronological and physiological aspects of aging. One challenge that we would like to acknowledge is the difficulty in separating interventions that impact the barrier directly from those that do so via other impacts on lifespan. To illustrate this point, it is entirely possible that activating a pro-apoptotic gene in the brain could lead to death accompanied by intestinal barrier failure. At the same time, we cannot exclude the possibility that similar relationships could exist between loss of function of any organ and general systemic health decline. For example, if there were an approach that would allow us to identify individual flies with impaired muscle function, it is possible that they would also show hallmarks of systemic aging. One challenge for the field is to better understand the cause-and-effect relationships between intestinal barrier dysfunction and systemic health decline. It will be interesting to further develop models of early-onset intestinal barrier dysfunction to provide novel insights.

In addition, many questions still exist surrounding the role of mitophagy/autophagy and mitochondrial health in maintaining the intestinal barrier. What are the molecular mechanisms that connect impaired mitochondrial health and intestinal dysfunction? Are tight junction components impacted? If so, how? Are inflammation and dysbiosis related to mitochondrial dysfunction? It will be especially interesting to understand the role of healthy mitochondria in intestinal health and to determine the specific molecular changes that accompany mitochondrial dysfunction and how they lead to alterations in intestinal function.

One major unanswered question involves whether mechanisms that accompany reversal of dysbiosis, inflammation and other aging markers will be useful to older organisms that are experiencing systemic deterioration. In other words, is it even possible to restore barrier function in older animals? Tight junctions in the fly begin to mislocalize in midlife, significantly prior to detectable intestinal barrier function as determined by the Smurf assay. It is too early to say whether interventions aimed at restoring barrier integrity in aged animals will be sufficient to overcome such long-term deterioration of the barrier. Furthermore, there are still questions about the detailed molecular mechanisms accompanying the increased lifespan that is induced by overexpression of a tight junction components, such as Ssk and IAP. It is clear that overexpression of Ssk leads to a decrease in intestinal permeability and dysbiosis, but details about how overexpressing just one of the tight junction equivalent components can lead to these changes need to be further evaluated. It could be that Ssk, which is known to be involved in localization and stability of the other junctional components, has a stronger impact through its overexpression than other junctional proteins, but further experiments need to be conducted to explore these questions. Another consideration is that the vertebrate tight junction is more complex than the invertebrate tight junction, with specific levels of numerous components important for maintaining barrier integrity. It will be important to gain a better understanding of each tight junction protein's role in maintaining the barrier in order to discover important targets for human interventions.

Ultimately, these various studies aim to extend human lifespans and health spans, ensuring a better quality of life as humans age. Much of the human data, however, are more difficult to obtain and not as clear. The human studies described within this Review are just the beginning, and reflect the types of studies that need to be expanded. It will be very interesting to gain more detailed knowledge about tight junction protein expression levels as humans age and their correlation with intestinal barrier integrity. It will also be very interesting to understand more about how these junctional proteins in humans change under different nutritional and exercise regimens or in individuals suffering from various health consequences, including inflammation and dysbiosis. There are many studies attempting to gain numerous details about human commensal bacteria and inflammation under various conditions, and it will be important to add information about intestinal barrier function and how specific tight junction proteins are altered under these conditions. Studies that measure levels of specific inflammatory cytokines combined with data from biopsied intestines revealing changes in tight junction levels, and data showing intestinal barrier function would be quite intriguing. The more data that are obtained from all organisms, including humans, the closer science can come to developing interventions that may help to postpone barrier dysfunction and extend human health and lifespans.
